# Phlegmasia Cerulea Dolens Secondary to Iliac Vein Compression From Posttransplant Hematoma Despite Anticoagulation

**DOI:** 10.31486/toj.25.0097

**Published:** 2026

**Authors:** Tiffany Xiu Zhen Lim, Godfrey R. Parkerson, Justin Barr

**Affiliations:** ^1^ The University of Queensland Medical School, Ochsner Clinical School, New Orleans, LA; ^2^ Department of Surgery, Ochsner Clinic Foundation, New Orleans, LA; ^3^ Multi-Organ Transplant Institute, Ochsner Clinic Foundation, New Orleans, LA

**Keywords:** *Blood coagulation disorders*, *homocysteine*, *kidney transplantation*, *methylenetetrahydrofolate reductase (NADPH2)*, *thrombophlebitis*, *venous thromboembolism*, *venous thrombosis*

## Abstract

**Background:**

Phlegmasia cerulea dolens is a rare, life- and limb-threatening form of venous thrombosis that is often seen in the setting of hypercoagulable states or mechanical obstruction.

**Case Report:**

We report the case of a 49-year-old female with a history of multiple thromboembolic events and a methylenetetrahydrofolate reductase mutation who, despite therapeutic anticoagulation, developed phlegmasia cerulea dolens following renal transplantation. Her postoperative course was marked by recurrent hemorrhage with rapidly reaccumulating subincision and perinephric hematomas, requiring 4 exploratory laparotomies for hematoma evacuation and graft evaluation. In addition, the patient underwent urgent endovascular suction thrombectomy for extensive iliofemoral thrombosis caused by extrinsic iliac vein compression.

**Conclusion:**

This case highlights the complex interplay between bleeding and clotting in high-risk transplant recipients and the need for nuanced anticoagulation management. Although phlegmasia cerulea dolens is rare, clinicians must keep a high index of suspicion for the pathology, as early recognition and timely intervention can be both life- and limb-saving.

## INTRODUCTION

Phlegmasia cerulea dolens represents the extreme end of the deep vein thrombosis spectrum and is characterized by extensive venous occlusion, limb swelling, cyanosis, and pain as the loss of outflow eventually compromises arterial inflow.^1,2^ Although rare, phlegmasia cerulea dolens is associated with a high risk for limb ischemia and death if not promptly recognized and treated.^1,2^ In the context of kidney transplantation, the incidence of thrombotic events such as phlegmasia cerulea dolens is heightened because of complex alterations in the hemostatic system that include both prothrombotic and hemorrhagic tendencies. These changes are driven by preexisting end-stage kidney disease, perioperative factors, and the effects of immunosuppressive therapy.^3^ The very nature of the transplant operation, including clamping the renal vein and possible venous compression by retractor blades, further elevates the risk.

Kidney transplant recipients with underlying hypercoagulable disorders face unique challenges because their need for perioperative anticoagulation increases bleeding risk while failing to protect fully against thrombotic events.

We present a case of phlegmasia cerulea dolens in a high-risk renal transplant recipient with a known methylenetetrahydrofolate reductase (MTHFR) mutation. MTHFR mutations are associated with an increased risk of pulmonary emboli and venous thromboembolism through disruption of folate and homocysteine metabolism, resulting in elevated homocysteine levels.^4,5^ Hyperhomocysteinemia shifts the hemostatic balance toward thrombosis by directly damaging endothelial cells, increasing oxidative stress by reducing nitric oxide bioavailability, activating factor V and factor XII, impairing protein C and antithrombin, and inhibiting fibrinolysis.^6,7^

To our knowledge, our case is only the second report of phlegmasia cerulea dolens in a kidney transplant recipient.^8^

## CASE REPORT

A 49-year-old female with end-stage renal disease secondary to focal segmental glomerulosclerosis underwent deceased donor renal transplantation. Her medical history was notable for hypertension, multiple prior pulmonary emboli, deep vein thromboses, and a known MTHFR mutation. She was maintained on therapeutic enoxaparin prior to transplant because she had previously developed pulmonary emboli on warfarin and apixaban. She had no relevant surgical history. The kidney, procured from a brain-dead donor with a kidney donor profile index of 16%, was sewn to the right external iliac artery and vein in the standard retroperitoneal position. Cold ischemia time was 1,009 minutes, and warm ischemia time was 28 minutes, for a total of 1,037 minutes. A Lich-Gregoir ureteroneocystostomy provided bladder drainage.

The patient's perioperative course was largely unremarkable. Hematology guided her anticoagulation management, and she was converted from a bridging heparin drip back to enoxaparin on postoperative day 3. She was discharged on postoperative day 4 with a functioning graft and no evidence of bleeding.

Three days postdischarge, the patient presented to the emergency department (ED) with severe right lower quadrant pain and near syncope. Her vital signs were stable without hypotension or tachycardia. Physical examination revealed abdominal tenderness and distension, along with tender nodular mass over the right upper gluteal and lower lumbar region without skin changes. The transplant incision site was nonerythematous and without drainage. Laboratory workup revealed white cell count of 13.32 K/μL (reference range, 3.9-12.7 K/μL), hemoglobin of 9.0 g/dL (reference range, 12.0-16.0 g/dL), and hematocrit of 27.7%. (reference range, 37.0%-48.5%), compared to the following posttransplant values at discharge: white cell count of 6.79 K/μL, hemoglobin of 11.0 g/dL, and hematocrit of 33.7%.

**Figure 1.**
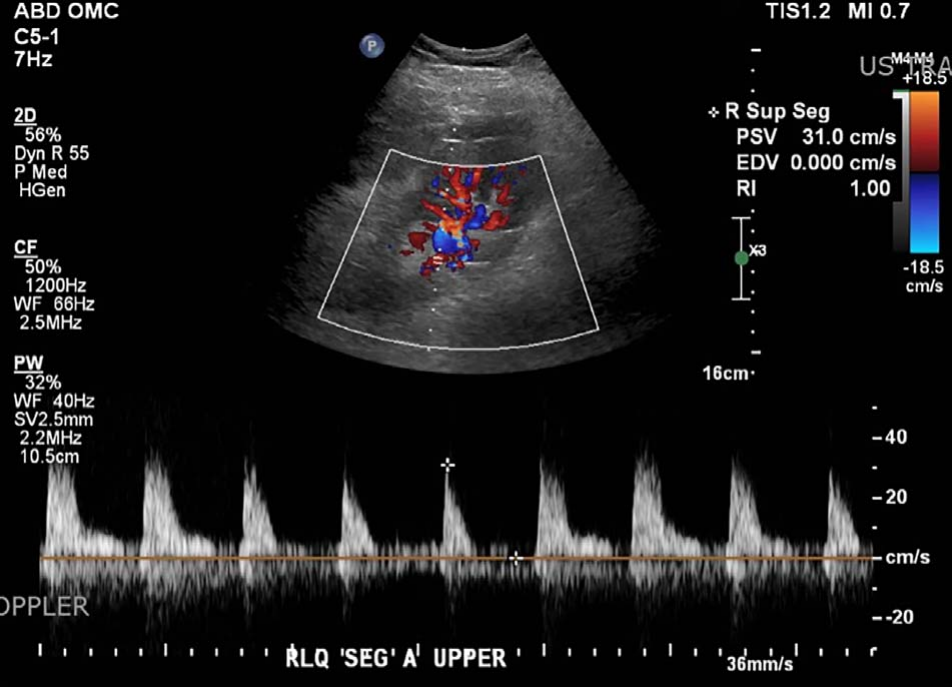
Doppler ultrasound of the transplanted kidney after the first exploration shows an elevated resistive index of 1.0 in an interlobar segmental artery with minimal diastolic flow. This image confirmed excellent inflow into the kidney but raised concerns about effective draining and/or swelling of the graft.

Computed tomography (CT) without contrast revealed a large subcutaneous and perinephric hematoma. Within 20 hours of her presentation to the ED, the patient underwent surgical evacuation of the hematoma; no evidence of active bleeding was seen. Doppler ultrasound immediately after the evacuation demonstrated a well-perfused kidney with excellent arterial waveforms but a resistive index of 1.0 and peak systolic velocity of 248 cm/s at the arterial anastomosis. The main renal vein flow was patent with a velocity of 20 cm/s, but the iliac vein could not be seen on imaging (Figure 1).

**Figure 2.**
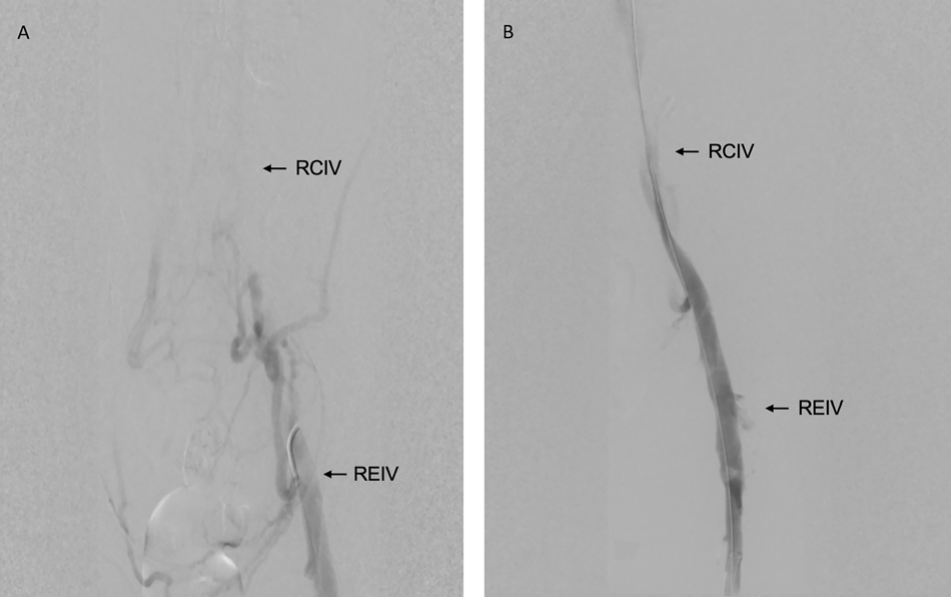
(A) Preintervention venogram shows diffuse collateral flow with minimal filling of the right external iliac vein (REIV) and no filling of the right common iliac vein (RCIV). (B) Postthrombectomy venogram shows improved filling of the REIV and reduced collateral flow but extrinsic compression of the RCIV.

Approximately 2 hours after completion of the surgical evacuation, the patient developed right lower extremity swelling, stiffness, and discoloration concerning for phlegmasia cerulea dolens. Ultrasound confirmed acute deep vein thrombosis in the right common femoral and proximal greater saphenous veins, prompting reinitiation of low-intensity heparin aiming for a partial thromboplastin time (PTT) of 45 to 60 seconds. Within 4 hours of symptom presentation and with the patient in the prone position, Vascular Surgery performed an emergent endovascular suction thrombectomy via ipsilateral popliteal vein access. Preintervention venogram with contrast demonstrated thrombus extending into the right common femoral vein and apparent occlusion of the common iliac vein (Figure 2A). Intraoperative intravascular venogram performed after thrombectomy revealed extrinsic compression of the common iliac vein by a new perinephric hematoma that was not present during the first surgical evacuation (Figure 2B). Notably, the renal vein was patent and draining through the internal iliac vein. Given the persistent venous compression following thrombectomy, the case was immediately handed over to the transplant surgery team for a second exploration and hematoma evacuation, after which the patient's abdomen was left open.

The phlegmasia cerulea dolens symptoms—lower extremity swelling, pain, and discoloration—began to improve quickly after thrombectomy and hematoma evacuation. However, the patient's PTT and heparin anti-Xa assay levels continued to rise, with PTT increasing from 78 seconds (reference range, 21.0-32.0 seconds) an hour after the second evacuation to >150 seconds 2 hours later, and heparin anti-Xa increasing from 1.16 IU/mL (reference range, 0.3-0.7 IU/mL) to >1.5 IU/mL. The patient was returned to the operating room 2 hours after the second set of PTT and heparin anti-Xa laboratory values were obtained for a third exploration because of bleeding due to supratherapeutic factor Xa levels. Two days after the third exploration, final exploratory laparotomy found no hematoma or active bleeding, a generally well-perfused kidney without diffuse retroperitoneal ooze, and no anastomotic issues. The patient's incision was closed.

Cultures from the first evacuated hematoma grew methicillin-resistant *Staphylococcus epidermidis, Staphylococcus hominis*, and *Enterococcus faecium*. The patient received 6 doses of intravenous linezolid 600 mg every 12 hours and then transitioned to oral linezolid 600 mg every 12 hours to complete a 14-day treatment course.

Despite these complications, the renal allograft remained viable, and the patient required only a single session of dialysis for acute kidney injury. She was discharged home after 12 days in the hospital and 4 surgeries. Her serum creatinine and glomerular filtration rate (GFR) on discharge from her kidney transplant were 1.4 mg/dL (reference range, 0.6-1.4 mg/dL) and 39 mL/min/1.73/m^2^ (reference value, >60 mL/min/1.73/m^2^), respectively. At discharge from the second admission, serum creatinine was 3.0 mg/dL, and GFR was 19 mL/min/1.73/m^2^. At her 4-month follow-up, serum creatinine was 1.6 mg/dL, and GFR was 39 mL/min/1.73/m^2^.

## DISCUSSION

In transplant recipients, the risk of venous thromboembolism is increased by factors such as malignancy, surgery and the subsequent decreased mobility, central venous catheters, and immunosuppressive therapy, while bleeding risk is heightened by perioperative factors, thrombocytopenia, platelet dysfunction associated with end-stage renal disease, and the use of anticoagulation.^9,10^ Intraoperative variables, such as surgical manipulation of the iliac vessels and surrounding tissues, can cause endothelial injury, which, when combined with a preexisting hypercoagulable state, increases the risk of thrombosis in kidney transplant recipients.^11,12^ Additionally, prolonged operative time and immobility during surgery contribute to venous stasis, compounding the risk of deep vein thrombosis.^13^

In kidney transplant recipients, the risk of deep vein thrombosis is markedly higher than in the general population, with cumulative incidences of 2.7% to 8.3% within the first 6 months, peaking in the first month posttransplant.^12,14,15^ By comparison, venous thromboembolism occurs in 0.5% to 3% of patients within 90 days of other high-risk major abdominal, pelvic, or oncologic surgeries.^16^ Among orthopedic patients, deep vein thrombosis rates are approximately 1.2% within 30 days and 1.9% within 90 days despite prophylaxis, increasing to 2.5% in individuals with thrombotic risk factors, such as hereditary hypercoagulability.^17^ Progression from deep vein thrombosis to phlegmasia cerulea dolens is uncommon, representing only a small fraction of thrombotic events.^2^

Phlegmasia cerulea dolens is a clinical diagnosis, with substantial limb swelling and pain as common presenting features, while cyanosis is pathognomonic. Duplex ultrasonography or CT with intravenous contrast may aid in diagnosis but is not required. As the disease progresses, bullae and venous gangrene may develop. Phlegmasia cerulea dolens most commonly involves thrombosis of the iliac and common femoral veins. Initial management includes limb elevation and fluid resuscitation to improve venous return, but many patients require more definitive therapy with catheter-directed thrombolysis, endovascular thrombectomy, and, rarely, open thrombectomy with ongoing anticoagulation. The decision to undergo thrombolysis or thrombectomy (endovascular vs open) depends on the extent of the thrombus, presence of venous gangrene, and refractory thrombosis despite anticoagulation.^2^ Without immediate identification and treatment, prognosis is poor, with mortality rates of 20% to 40%.^18^ Prevention strategies mirror those for deep vein thrombosis and include pharmacologic prophylaxis, mechanical measures such as sequential compression devices and leg elevation, adequate hydration, and early mobilization.

To our knowledge, no studies have quantified the incidence of phlegmasia cerulea dolens in kidney transplant recipients, and only 1 case report has been published. In 1995, Killewich et al described a patient who developed phlegmasia cerulea dolens involving the bilateral external and common iliac veins and the left femoral vein 5 months after kidney transplantation; the patient was successfully treated with urokinase, resulting in allograft preservation.^8^ In contrast, our patient developed phlegmasia cerulea dolens within 1 week of transplantation, and the process was unilateral, precipitated by extrinsic compression from recurrent perinephric hematomas in the setting of a hypercoagulable state. Because our patient was within 30 days of major surgery and had recurrent perigraft hemorrhage, she had absolute contraindications to thrombolytic therapy. Instead, she underwent preintervention venogram, intravascular venogram, and endovascular thrombectomy. While the patient reported by Killewich et al^8^ and our patient achieved graft salvage, our patient experienced a decline in renal function, highlighting the potential for partial but not complete recovery of the renal graft at discharge. We postulate that the patient's transplanted kidney survived despite extensive surrounding thrombosis because outflow was maintained through retrograde cross-pelvic flow via the internal iliac vein, and prompt suction thrombectomy and therapeutic anticoagulation prevented complete occlusion.

This case adds to the limited literature describing phlegmasia cerulea dolens in kidney transplant recipients and highlights the complex interplay between thrombosis and bleeding in this population.

## CONCLUSION

To our knowledge, this case is only the second reported case of phlegmasia cerulea dolens in a kidney transplant recipient. The case emphasizes the importance of early recognition and prompt intervention for phlegmasia cerulea dolens, particularly when anatomic factors such as hematomas create venous outflow obstruction. Careful multidisciplinary assessment and individualized management are essential to optimize outcomes in this high-risk population.

## Notes

*This article meets the Accreditation Council for Graduate Medical Education and the American Board of Medical Specialties Maintenance of Certification competencies for Patient Care and Medical Knowledge.*
